# The Nervous Manifestations of Rickets

**Published:** 1908-07-04

**Authors:** James Burnet


### SPECIAL ARTICLE.

### THE NERVOUS MANIFESTATIONS OF RICKETS.

By JAMES BURNET, M.A., M.D., M.R.C.P. Ed.

Beyond the restlessness and tenderness, which are two of the earliest evidences of rickets, there are certain important manifestations which must be carefully kept in mind. Of these the best known is convulsions. A convulsive seizure occurring during the first year of life is nearly always due to rickets. It may, however, be a premonitory symptom of the onset of some infectious disease, or may be induced by the presence of a loaded colon or by some dietetic error. In the majority of cases, however, rickets is found to be present in the patient suffering from convulsions. The nervous system during the period of infancy is very unstable, and is in consequence very readily upset. This is more especially the case in rachitic subjects. It must not be forgotten, however, that convulsions in infancy may prove to be the forerunners of true epileptic seizures in later life. Consequently infantile convulsions should always be regarded seriously, and treated with great care and watchfulness.

Another nervous manifestation of rickets is what is known as laryngismus stridulus. In this condition the child often catches his breath, or breathes somewhat stridulously with a sharp, crowing sound. This condition is apt to prove very alarming, and may give rise to the impression that the child is the subject of a laryngitis, or even of diphtheria. Sometimes the laryngismus is not at all marked unless the infant is excited or crying. When not present it is occasionally brought on by examining the throat. The tongue depressor used in this process sets up irritation, and brings on the crowing sound. In itself laryngismus is not a serious affection, but it is very liable to be associated with convulsive seizures, and, moreover, it leads in many instances to marked interference with the infant's respiration.

Tetany is a third important evidence of rickets. The symptoms of this condition are too well known to require description. It often happens that the hands and feet are swollen, and this appearance has been sometimes mistaken for renal dropsy. In our experience many of these cases are the result of dilatation of the stomach. It is a well-known fact that in rickets the colon is apt to become dilated, and as a result we get distension of the abdomen. The stomach likewise may become dilated in this disease, and so give rise to the condition of tetany. Whether this is so in every case we are not prepared to say, but in many cases of tetany coming under our immediate observation we have found on auscultatory percussion that the stomach was considerably dilated. Gastrectasis as a cause of tetany is worth keeping in mind, as this condition of the stomach is readily brought about by overloading it with unsuitable, and especially with fermenting starchy food, which is a certain cause of rickets.

Facial irritability is a common evidence of rickets, and this sign may be present when all other nervous manifestations are lacking. It may be brought out by tapping over the masseter, when characteristic facial movements are occasioned. Infants in whom this sign is present are liable to suffer from convulsions. It may therefore be regarded as a danger signal and as a measure of the nervous stability of the patient. The more marked the facial irritability is found to be, the more likely is the infant to suffer from some of the other nervous phenomena of rickets.

Head-rolling, with or without nystagmus, is commonly found in association with rickets. This condition is generally stated to be the result of living in a dark dwelling. This, however, has so far not been our experience. Head-rolling is met with in children who live in airy, bright rooms as well as in those who spend the greater part of their existence in badly ventilated and dark houses. This phenomenon is not very readily explained. It is, of course, due to nervous instability, but why some rachitic infants should show it while others do not we cannot profess to explain. It usually passes off before the eighteenth month.

As to the treatment of these nervous manifestations, there are certain important points which must be carefully borne in mind. In the first place, cow's milk must be substituted for condensed milk and artificial foods. Babies fed on these are more liable than others to display these nervous phenomena. Where breast-feeding is impossible cow's milk suitably diluted and unsterilised forms the only real substitute. Then, again, cod-liver oil is always beneficial. Petroleum is absolutely valueless in such cases, as being a mineral oil, it cannot possibly be absorbed. Very important, too, is cold douching of the spine. This may be carried out immediately after the baby has been bathed. A sponge or jug of cold water is poured down his back, which is then quickly dried. If this is done before a fire there is no risk whatever of catching cold. Sedatives, such as bromides, phenazonum and the like should be avoided as a rule. They are generally unnecessary, even for the treatment of convulsions and tetany. Fresh air is also essential, both by day and by night, and the patient should be taken out every forenoon and afternoon, and, if possible, kept outside altogether during the day, provided the weather is favourable.

